# Draft genome sequences of *Azospirillum argentinense* Ap18 and *Gluconacetobacter sacchari* Ap92 isolated from sugarcane roots in Colombia

**DOI:** 10.1128/mra.00233-25

**Published:** 2025-08-25

**Authors:** Sergio Pardo-Díaz, Julian Másmela-Mendoza, Paola Delgadillo-Duran, Eddy J. Bautista, Daniel F. Rojas-Tapias

**Affiliations:** 1 Corporación Colombiana de Investigación Agropecuaria-AGROSAVIA, Bogotá, Cundinamarca, Colombia; 2 Colombian Sugar Cane Research Center-Cenicaña 42703 , Florida, Valle del Cauca, Colombia; University of Strathclyde, Glasgow, United Kingdom

**Keywords:** plant growth-promoting bacteria, sugarcane, *Gluconacetobacter*, *Azospirillum*

## Abstract

We report the draft genomes of *Azospirillum argentinense* Ap18 and *Gluconacetobacter sacchari* Ap92, which were isolated from sugarcane roots in the Cauca River Valley in Valle del Cauca, Colombia. The genomes are 7,412,287 bp and 4,484,949 bp in length, with 6,695 and 4,199 predicted coding sequences, respectively.

## ANNOUNCEMENT

Plant microbiome refers to the community of microorganisms associated with plants, playing roles in plant growth and development (1). Harnessing this microbiome represents a promising and environmentally friendly strategy for enhancing crop productivity (2); for instance, species of *Azospirillum* (3–5) and *Gluconacetobacter* (6, 7) have demonstrated potential to enhance plant growth. We report the draft genomes of *Azospirillum argentinense* Ap18 and *Gluconacetobacter sacchari* Ap92.

Ap18 and Ap92 were isolated from sugarcane roots in 2015 in the Cauca River Valley in Valle del Cauca, Colombia, using semi-solid NFb and LGI-P media, followed by purification in Congo red plates (8). We obtained both strains, Ap18aSsH-2.2 and Ap92ScSc-25.2, from Cenicaña’s (Colombia) culture collection, where they are preserved at −80°C. Cells were reactivated from frozen stocks and cultured in DYGS medium at 30°C for 24  h. We extracted genomic DNA using the PureLink Genomic DNA Mini Kit (Invitrogen); integrity and concentration were verified by electrophoresis and Qubit dsDNA BR Assay Kit (Invitrogen). Libraries were prepared using the NEBNext Ultra II FS DNA Library Prep Kit for Illumina (NEB). For each strain, two libraries were identically prepared from the same gDNA extraction. Sequencing was performed using NovaSeq X Plus (Azenta, USA), generating 2 × 150 bp PE reads. Read quality was determined using FastQC v.0.12.1 (9), and adapter removal and low-quality base trimming with Trimmomatic v.0.39 (10). Reads from two identical libraries of each strain were combined post-trimming. Genome assembly was performed using SPAdes v.3.15 (11) and quality assessed with QUAST v.5.3.0 (12). Contigs <1000 bp were discarded. Contaminant sequences were filtered out if they showed <30% identity to a local database constructed from *Azospirillum* or *Gluconacetobacter* RefSeq genomes (13). Excluded contigs were confirmed as non-bacterial via BLASTn (nr database). Annotation was performed using PGAP v.6.9 (14). TYGS v400 was used for whole genome-based taxonomic analysis and digital DNA-DNA hybridization (dDDH; formula d_4_) (15). Completeness and contamination were assessed using BUSCO v.5.8.3 (16) and CheckM v.1.2.3 (17). Coverage was calculated using BWA v.0.7.19 (18) and SAMtools v.1.21 (19). Default parameters were used for all software.

*A. argentinense* Ap18 genome comprised 162 contigs (N_50_ = 97.1 kb, L_50_ = 23). The %GC content was 68.5%. The genome was 7,412,287 bp in size, with a sequencing coverage of 65×, and was assembled from 1,701,370 trimmed PE reads. The largest contig was 285,237 bp. Completeness was 99.7% using BUSCO (*rhodospirillales_odb10* database) and 95.6% (contamination 9.8%) using CheckM. The genome contained 6,695 CDSs, 6,642 protein-coding genes, and 69 tRNAs. Whole genome-based taxonomy revealed that Ap18 has a dDDH of 86.6% with *A. argentiniense* Az39^T^ (GCF_000632475.1).

*G. sacchari* Ap92 genome comprised 134 contigs (N_50 _=71.8 kb, L_50_=17). The %GC content was 66.0%. The genome was 4,484,949 bp in length, with a sequencing coverage of 92×, and was assembled from 1,469,235 trimmed PE reads. The largest contig was 243,335 bp. BUSCO showed a completeness of 99.2% (*rhodospirillales_odb10* database) and CheckM of 91.12% (contamination 6.72%). The genome contained 4,199 CDSs, 4,092 protein-coding genes, and 46 tRNAs. Whole genome taxonomic analysis showed that Ap92 has a dDDH of 92.4% with *G. sacchari* LMG 19747^T^ (GCF_014174255.1).

## Data Availability

The genome sequences of *Azospirillum argentinense* Ap18 and *Gluconacetobacter sacchari* Ap92 are available under bioproject PRJNA1190442 with accesion numbers JBJLSN000000000.1 and JBKQXQ000000000.1, respectively. The raw reads for all libraries were deposited with SRA accession numbers SRR32483262 and SRR32483261.
